# Peripheral sentinel lymphadenectomy in 163 dogs: Postoperative surgical complications and comparison between intraoperative dissection techniques

**DOI:** 10.1111/vsu.14246

**Published:** 2025-03-17

**Authors:** Giovanni Mattioli, Marzia Cino, Damiano Stefanello, Dario Drudi, Emanuela Maria Morello, Guido Pisani, Lavinia Elena Chiti, Alessio Pierini, Elisa Maria Gariboldi, Donatella De Zani, Federico Massari, Davide Giacobino, Marina Martano

**Affiliations:** ^1^ Department of Veterinary Medical Science University of Parma Parma Italy; ^2^ Centro Veterinario Pisani‐Carli‐Chiodo Luni Mare Italy; ^3^ Department of Veterinary Medicine and Animal Sciences University of Milan Lodi Italy; ^4^ Clinica Veterinaria Nervianese Milan Italy; ^5^ Department of Veterinary Science University of Turin Grugliasco Italy; ^6^ Clinics for Small Animals Surgery, Vetsuisse Faculty University of Zurich Zurich Switzerland

## Abstract

**Objective:**

The first aim was to describe the incidence and severity of surgical complications following peripheral lymphadenectomy in dogs. The second was to compare three surgical techniques: unassisted lymphadenectomy, intraoperative guidance by methylene blue dye alone (MB) or by a combination of γ‐probe and MB (γ‐MB). The third was to assess whether the number, palpability, and site of lymph nodes (LNs) influenced the incidence of complications.

**Study design:**

Retrospective multicenter study.

**Sample population:**

Lymphadenectomies (*n* = 201) from 163 client‐owned tumor‐bearing dogs.

**Methods:**

Medical records of dogs undergoing both preoperative sentinel LN (SLN) mapping and excision of peripheral SLNs between December 2020 and April 2023 were reviewed. Signalment, intraoperative assistance technique, number of LNs, surgical time, postoperative treatments, site, and timing of complications observed were collected.

**Results:**

Seventy‐two (36%) lymphadenectomies were performed without assistance, 24% with MB and 40% with γ‐MB. The overall incidence of surgical complications was 7.5%, of which 80% were mild. The most frequent complication was seroma (2.5%). None of the variables considered in the logistic regression model, including intraoperative guidance, influenced the complication rate (*p* = .255). Using the decision tree statistical model, mandibular and retropharyngeal lymphadenectomy affected the complication rate when surgery lasted more than 21.5 min.

**Conclusion:**

Lymphadenectomy of peripheral LNs was associated with a low rate of mild complications, regardless of intraoperative assistance. Mandibular and retropharyngeal lymphadenectomies lasting more than 21.5 min may result in more complications.

**Clinical significance:**

Lymphadenectomy of superficial LNs is a safe procedure that is easy to perform in most cases, even without intraoperative assistance.

**Abbreviations**
99mTc: technetium 99 metastable
BCS: body condition score
CEUS: contrast‐enhanced ultrasound
ICTL: indirect computed tomographic lymphography
LNs: Lymph nodes
LS: lymphoscintigraphy
LT: time for lymphadenectomy
MB: methylene blue dye
MCTs: mast cell tumors
NIRF: near‐infrared fluorescent lymphography
NSAIDs: nonsteroidal anti‐inflammatory drugs
RLN: regional lymph node
SLN: sentinel lymph node
γ‐MB: γ‐probe and methylene blue dye

## 1 INTRODUCTION

Assessing the neoplastic involvement of lymph nodes (LNs) is essential to determine the spread of a tumor, to determine the need for adjuvant treatment, and to accurately predict prognosis in both canine and feline cancer patients.^1^, ^2^, ^3^, ^4^, ^5^, ^6^, ^7^, ^8^, ^9^ The identification of the draining LN—the so‐called “sentinel lymph node” (SLN)—has recently gained increased attention in veterinary oncology, especially for tumors that preferentially metastasize to the LNs, such as mast cell tumors, oral malignant melanomas, and other epithelial malignancies.^10^, ^11^, ^12^, ^13^ It has been suggested that, especially in tumors with a high propensity for LN metastasis, detecting and excising SLN may improve the outcome. Cytology may not always clearly distinguish metastatic from nonmetastatic LNs, as early lymph‐node metastasis may be missed, as reported, for example, in canine mast cell tumors (MCTs).^14^, ^15^ Sentinel lymph node mapping is therefore considered important for comprehensive staging.

Numerous studies have enriched the literature with various protocols for LN detection, both preoperatively and intraoperatively, reporting the detection rate and the agreement between regional (RLN) and SLN in different tumors.^2^, ^10^, ^16^, ^17^, ^18^, ^19^ Thus, the number of lymphadenectomies has increased in recent years in veterinary medicine. Reported mapping techniques vary widely, from more clinically and financially accessible methods —indirect radiographic lymphography, indirect computed tomographic lymphography (ICTL), contrast‐enhanced ultrasound (CEUS)—to more sophisticated ones, namely lymphoscintigraphy (LS) and near‐infrared fluorescent lymphography (NIRF).^10^, ^16^, ^17^, ^18^, ^19^, ^20^, ^21^, ^22^ The intraoperative identification of LN can be challenging. The use of intraoperative assistance, such as intraoperative gamma‐probing (γ‐probing) after the injection of technetium 99 metastable (99mTc), fluorescent lymphography with indocyanine green, or methylene blue dye (MB) has begun to be used during SLN resection in referral veterinary hospitals.^2^, ^5^, ^12^, ^23^, ^24^, ^25^

A low complication rate for sentinel lymphadenectomy in dogs has been reported,^1^, ^10^, ^20^, ^23^, ^24^, ^25^, ^26^, ^27^ but few studies specifically compared the surgical complication rate with different intraoperative dissection techniques for lymphadenectomy.^2^, ^24^, ^25^

The first aim of this study was therefore to describe the incidence and the severity of surgical complications following peripheral lymphadenectomy in dogs. The second aim was to compare three surgical techniques: unassisted lymphadenectomy, intraoperative guidance by methylene blue dye alone (MB), or by γ‐probe associated with MB dye (γ‐MB). The third aim was to assess whether the number, palpability, and the site of SLNs influenced the incidence of complications. All the SLNs were identified by preoperative LS or ICTL. The authors hypothesized that preoperative identification of the lymphocentrum—defined as “one or a group of LNs in the same region receiving afferent vessels from approximately the same area”^28^—would reduce the risk of complications when intraoperative assistance was used and when LNs were fewer in number, palpable, or located in specific anatomical sites (popliteal, inguinal, or superficial cervical).

## 2 MATERIALS AND METHODS

Medical databases of three veterinary teaching hospitals and two private veterinary clinics were searched retrospectively for client‐owned dogs that underwent both preoperative SLN mapping and lymphadenectomy between December 1, 2020, and April 30, 2023. Although lymphadenectomy might be performed for different reasons, only those for SLN excision surgery were included in the study as it was the most common procedure performed by the authors. To identify SLN, each institution used one of two preoperative mapping techniques: ICTL or LS. Dogs were eligible for inclusion in this study if peripheral SLN excision was performed using one of the three surgical techniques—unassisted lymphadenectomy, intraoperative guidance by methylene blue dye alone (MB) or by the combination of γ‐probe and MB (γ‐MB)— regardless of the preoperative detection method used. Based on the preoperative mapping and intraoperative assistance techniques, the population was divided into three groups:

1. Preoperative LS and intraoperative γ‐MB
2. Preoperative ICTL and intraoperative MB
3. Preoperative ICTL and unassisted lymphadenectomy

Further inclusion criteria were a minimum follow up of 30 days and complete information about postoperative treatment and any surgical complications.

Exclusion criteria were intracavitary LNs, cases where all lymphocenters draining the same anatomic district were excised concurrently (e.g., simultaneous bilateral removal of mandibular and retropharyngeal SLNs), megalic SLNs, and cases without recorded time for lymphadenectomy (LT). Lymph nodes were defined as megalic when severely enlarged, disrupting the normal regional anatomy, and/or adhering to surrounding tissues; these were excluded as this is not a typical clinical scenario and it may have introduced a bias in both the time needed to excise them and the complication rate.

All owners signed written informed consent prior to the surgical procedure, and consent for their dogs’ data to be used for scientific purposes. All the procedures were conducted as part of the standard treatment protocol for oncologic diseases, following the institutional guidelines for animal welfare, under the control of the National Ministry of Public Health. No extra animal discomfort was caused for the purpose of the study.

Lymphadenectomies with the assistance of a γ‐probe (Crystal Probe SG04, Crystal Photonic GmbH, Berlin, Germany) associated with MB were performed by the same three surgeons at one institution following preoperative LS. The decision to use MB (0.4 mL of 5 mg/mL sterile methylene blue) (SALF S.p.A, Cenate Sotto, Bergamo, Italy) alone or to perform unassisted procedures following the detection of the SLN by ICTL was based on the surgeon's preference in the remaining four institutions but was not related to the anatomic site of the lymphocentrum.

Data retrieved for each patient included breed, sex, age, body weight, body condition score (BCS) (range 1–9), anatomic location, size, and laterality of the SLNs, and whether they were clinically palpable during physical examination. Lymph nodes were defined as palpable if detectable by manual palpation of the anatomic area in which they were located. Types of preoperative mapping technique (LS or ICTL), whether intraoperative guidance for LN excision was performed, and the type of guidance (colorimetric identification by MB or a combination of MB and handheld γ‐probe) were registered. The number of excised LNs and their maximum diameter (measured ex vivo with a caliper after surgical resection), time to perform the lymphadenectomy (LT), postoperative treatment(s), number and type of intraoperative and postoperative complications observed, and any further surgical intervention or medical treatment were recorded. If only one incision was performed to remove both the tumor and the lymphocentrum, lymphadenectomy was measured from the start of the search for the lymphocentrum. If the approach to the lymphocentrum required a skin incision different from the one used to remove the primary tumor, the LT was recorded from the skin incision over the lymphocentrum until the skin closure. If several lymphocenters had to be removed from different sites, LT was recorded separately for each center. Dogs were clinically evaluated 3, 7, and 14 days after surgery, or at different intervals when owners detected any wound alteration.

The surgical complications evaluated included infection, dehiscence, seroma (localized collection of fluid at the site of tissue removal or other surgical procedures),^26^ hematoma, lymphoedema (a swelling in the wound area caused by loss of lymph fluid into the surrounding tissue),^26^ lameness, hemorrhage, and brachial plexus injury (BPI). The diagnosis of seroma was obtained clinically by palpation, or by ultrasound‐guided needle aspiration in case of doubt regarding the presence of fluid.

Surgical complications were defined and graded into five grades of severity: mild (grade 1), moderate (grade 2), severe (grade 3), life threatening (grade 4), and death (grade 5), as described by LeBlanc et al.^29^ For each complication, the onset (days after surgery), duration (days until complete resolution), and required treatment were recorded.

In the case of the excision of axillary LNs, thoracic limb reflexes were evaluated before and after surgery and any alteration was recorded, due to the possibility of BPI. In these dogs, pre‐existing lameness was recorded and reassessed after surgery.

For statistical purposes, each dissected lymphocentrum was considered a single case if the lymphadenectomy required a different skin incision on the same dog. Bilateral lymphadenectomy was considered as a single case when the LNs were removed from the same skin incision (for example, mandibular, or retropharyngeal LNs).

All preoperative mapping procedures (ICTL and LS) were performed under general anesthesia. Dogs were premedicated using different drugs at the discretion of the anesthesiologist. Computed tomographic images were acquired using a 16‐slice multidetector computed tomography (MDCT) unit (Siemens Somatom Go, Milan, Italy), a 20‐slice MDCT unit (Siemens Somatom Sensation Open, Munich, Germany), or a 16‐slice MDCT unit (Siemens Somatom Emotion 16, Erlangen, Germany), depending on institutional availability. A total of 1 mL of undiluted Iomeprole (Iomerson 300, Bracco, Italy), with an iodine concentration of 300 mg/mL, was injected peritumorally in all ICTLs.

All LSs (Prism 2000 XP; Picker International, Highland Heights, Ohio) were performed by injecting 99mTc ‐labeled nanosized human serum albumin (Nanoalbumon; Radiopharmacy Laboratory Ltd., Budaörs, Hungary) in four quadrants at a dose of 7.8–31 MBq/0.5 mL.

### 2.1 Statistical Analysis

Statistical analysis was conducted using R (version 4.0.5) for Windows (R Core Team, 2013), available under the General Public License. The logit model was implemented using the “glm2” package (version 1.2.1) from CRAN, and the decision tree analysis was performed using the “tidymodels” package (version 1.2.0) from CRAN. The normality of continuous data was assessed by the Shapiro–Wilk test. Continuous variables were expressed as medians and ranges in the case of non‐normal distributions, and as means ± standard deviations (SD) in the case of normal distributions. The analysis was applied in two steps: the first aimed to investigate the correlation between the presence of surgical complications and signalment, clinical presentation, LT, intraoperative LN excision technique, number, anatomic location, maximum diameter, palpability, and side of LNs removed using a logistic regression model. Univariate analysis was then used to assess if the presence of at least one complication was influenced by the use and the type of LN excision technique. Significance was set at *p* < .05. As a second step, a decision tree algorithm was applied, identifying a threshold in the continuous or discrete variables below and above which the probability of showing a complication became significantly lower or higher compared with the probability of not developing any complication.

## 3 RESULTS

The study included 201 peripheral lymphadenectomies performed on 163 dogs. The median age of enrolled dogs was 8 years (range 2–16 years), and the mean body weight was 24.8 kg (range 3–55 kg, SD 12.02). Data regarding breed, sex, and BCS are listed in Supporting Information, Table S1.

Table 1 reports the characteristics of excised LNs (anatomic location, laterality, and whether they were palpable). According to physical examination, 58 (28.9%) LNs were considered clinically palpable, of which 22 were excised without assistance, 18 with MB alone, and 18 with γ‐MB; 143 LNs (71.1%) were nonpalpable, of which 50 were excised without assistance, 31 with MB alone, and 62 with the assistance of γ‐MB. Of the 58 palpable LNs, 16 (27.6%) were superficial cervical LNs, six (10.3%) were axillary LNs, 19 (32.8%) were popliteal LNs, and 17 (29.3%) were mandibular LNs. Of the 143 nonpalpable LNs, 70 (49%) were inguinal, 38 (26.6%) were axillary, 22 (15.3%) were superficial cervical, seven (4.9%) were preaxillary, five (3.5%) were retropharyngeal, and one (0.7%) was a parotid LN.

**TABLE 1:** Characteristics of the excised lymph nodes.

Categorical variable	Frequency	%
Anatomic location		
Inguinal	70	34.8
Axillary	44	21.9
Preaxillary	7	3.5
Superficial cervical	38	18.9
Popliteal	19	9.4
Mandibular	17	8.5
Retropharyngeal	5	2.5
Parotid	1	0.5
Laterality		
Unilateral left	88	43.8
Unilateral right	107	53.3
Bilateral	6	2.9
Palpable	58	28.9
Nonpalpable	143	71.1

The median number of excised LNs was one (range 1–4 LNs). In 16 (7.9%) cases, more than two LNs were extirpated from a single lymphocentrum. The median maximum diameter of the LNs was 15 mm (range 1–49 mm). The mean LT was 18.6 min (range 1.5–83 min, SD 12.2).

No intraoperative complications occurred. Of the 201 surgical procedures performed, 93.1% (*n* = 187) did not lead to the development of any surgical postoperative complications and 6.9% (*n* = 14) did. The overall incidence of postoperative surgical complications was 7.5% (15/201). The most frequent was seroma at the surgical site, which developed in 5/201 (2.5%) cases, followed by lymphoedema in three cases (1.5%), dehiscence in three cases (1.5%), lameness in three cases (1.5%), and hematoma in one case (0.5%).

Time to onset and resolution of complications was available in all cases. The median time to onset of the surgical complication was 3 days (range 1–15 days), and the median duration was 4 days (range 1–10 days). Of the 15 surgical complications, 12 (80%) were mild (grade 1) and self‐limiting, and 3 (20%) were moderate (grade 2) and required medical intervention. No severe (grade 3), life‐threatening (grade 4), or fatal (grade 5) surgical complications were recorded.

The median time to onset of the seroma was 7 days (range 3–15 days) and the median duration was 6 days (range 4–10 days). It was classified as grade 1 in three cases and grade 2 in two cases, which required treatment with prolonged administration of NSAIDs. Two cases of suture dehiscence occurred 7 days after surgery and both resolved in 4 days without treatment (grade 1); one case occurred 4 days after surgery and was treated with a primary closure with skin staples (grade 2). All three cases of lymphoedema were classified as grade 1 and were self‐limiting. Hematoma and lameness were also self‐limiting. The three cases that developed transient lameness underwent axillary lymphadenectomy. Neurologic complications (e.g., permanent nerve injury), hemorrhage, and infection were not observed in any patient.

Preoperative ICTL was performed in 121 (60.2%) cases; of those, 72 (59.5%) were followed by lymphadenectomy without assistance, and 49 (40.5%) by peritumoral injection of MB. In 80 (39.8%) lymphadenectomies, preoperative LS and intraoperative γ‐MB dye were used (Table 2).

**TABLE 2:** Patient characteristics, physical examination findings, lymph node characteristics, duration of lymphadenectomy, and postoperative complications classified by lymph node localization techniques.

Variable	Unguided (*n* = 72)	MB (*n* = 49)	γ‐MB (*n* = 80)
Median body weight (kg)	29.2	28	27
First quartile	22	12	11
Third quartile	34	35	35
Median body condition score (1–9)	4	5	5
First quartile	4	5	5
Third quartile	5	5	5
Palpable (*n*)	22 (30.6%)	18 (36.7%)	18 (22.5%)
Nonpalpable (*n*)	50 (69.4%)	31 (63.3%)	62 (77.5%)
Median lymph node diameter (mm)	14	10	16
First quartile	10	10	12
Third quartile	20	25	25
Lymph node excised (*n*)			
<2	69 (95.8%)	42 (85.7%)	74 (92.5%)
>2	3 (4.2%)	7 (14.3%)	6 (7.5%)
Lymph nodes excised (median)	1	1	1
First quartile	1	1	1
Third quartile	1	1	1
Median duration of lymphadenectomy (min)	13.5	13	16.5
First quartile	10	10	12
Third quartile	19	20	21
Accessory axillary	2 (2.7%)	0	5 (6.2%)
Axillary	15 (20.8%)	16 (32.7%)	13 (16.3%)
Inguinal	29 (40.3%)	17 (34.7%)	24 (30%)
Mandibular	4 (5.6%)	2 (4.1%)	11 (13.7%)
Parotid	1 (1.4%)	0	0
Popliteal	8 (11.1%)	4 (8.1%)	7 (8.8%)
Retropharyngeal	2 (2.8%)	1 (2.1%)	2 (2.5%)
Superficial cervical	11 (15.3%)	9 (18.3%)	18 (22.5%)
complications (n)	2 (2.7%)	6 (12.2%)	6 (7.5%)

Abbreviations: MB, methylene blue dye alone; γ‐MB, γ‐probing and methylene blue dye.

Surgical complications occurred after lymphadenectomy of the inguinal lymphocentrum in 4/70 (5.7%) cases, mandibular lymphocentrum in 2/17 (11.8%) cases, axillary lymphocentrum in 3/44 (6.8%) cases, preaxillary lymphocentrum in 0/7, superficial cervical lymphocentrum in 3/38 (7.8%) cases, retropharyngeal lymphocentrum in 2/5 (40%), popliteal in 0/19 cases and parotid in 0/1. Of the lymphadenectomies that resulted in complications, one was bilateral (1/6), seven left sided (7/89), and six right sided (6/106). Complications occurred in 6.3% (9/143) of the lymphadenectomies of nonpalpable LNs and in 8.6% (5/58) of palpable LNs; in 12/185 cases when two or less LNs were removed (6.4%), and in 2/16 when three or more LNs were removed (12.5%). Three dogs with BCS = 4, five with BCS = 5, four with BCS = 6, and one with BCS = 7 developed complications. Six of 49 (12.2%) lymphadenectomies performed with MB assistance developed a total of six surgical complications (three instances of lameness, two seromas and one hematoma); six of 80 (7.5%) lymphadenectomies performed with γ‐MB developed a total of seven surgical complications (one dehiscence, three seromas, and three lymphedemas); two of 72 (2.7%) unassisted lymphadenectomies developed a total of two dehiscences.

In the logistic regression model, when considering the presence or absence of surgical complications as the dependent variable, body weight (*p* = .079), sex (*p* = .784), LN location (*p* = .885), laterality (*p* = .855), size of LNs (*p* = .779) and number of LNs removed (*p* = .453), assistance technique (*p* = .255) and LT (*p* = .631) were identified as nonsignificant factors and did not influence the occurrence of complications. Univariate analysis was used to assess whether the presence of at least one complication was influenced by one of the surgical techniques used, and no significant difference was found (*p* = .255).

**FIGURE 1:**
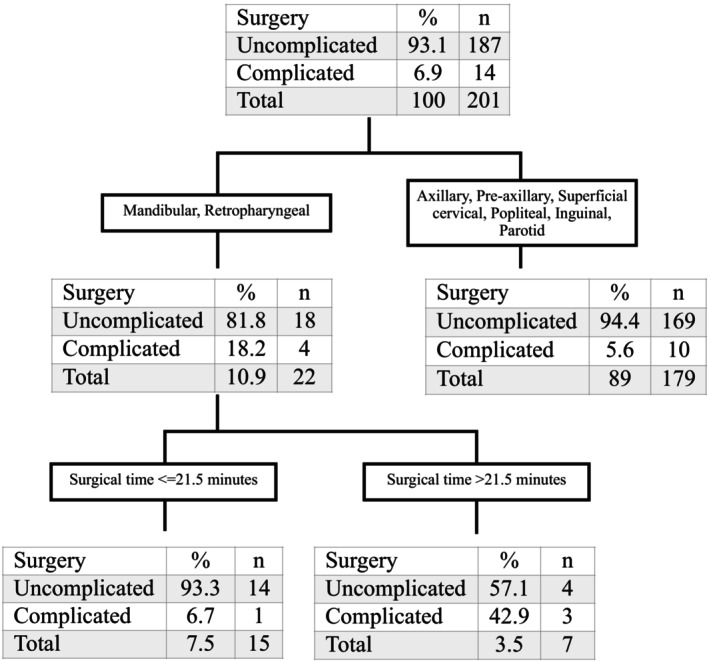
The decision tree model identified two thresholds that significantly increased the probability of developing at least one complication.

The second step of the statistical analysis was based on the decision tree model. Two nodes of the decision tree had a significantly higher incidence of complications than the others. These nodes were the LN anatomic site (mandibular and retropharyngeal) and the LT for this anatomic site (Figure 1). The highest incidence of complications was reported for mandibular and retropharyngeal lymphadenectomies (4/22), in comparison with 10/179 for preaxillary, axillary, superficial cervical, popliteal, inguinal, and parotid lymphadenectomies, which also had complications. A surgical time greater than 21.5 min increased the risk of complications after mandibular and retropharyngeal lymphadenectomy.

## 4 DISCUSSION

The first aim of this study was to describe the incidence and the severity of surgical complications following peripheral lymphadenectomy in dogs. Most of the lymphadenectomies performed at the institutions included were performed for SNL excision so, to obtain a homogeneous sample, only those cases were included. Indeed, when the sentinel lymphocentrum has been identified preoperatively, the surgical procedure, and hence the likelihood of developing surgical complications, is the same for any procedure, regardless of the cause for which it is performed. The results of this study can therefore be considered valid for any peripheral lymphadenectomy in the dog. An exception may be made for severely enlarged (megalic) LNs, as can be found in the advanced stages of tumor development, because the invasion of the LN capsule may induce adhesions to surrounding tissues and a subsequent need for wider and more disruptive surgery—hence the decision to exclude those cases from the study.

The failure to detect and remove one or more SLNs could result in downstaging of the patient. Intraoperative detection techniques are therefore employed commonly to improve the success rate of lymphadenectomies, expedite LN localization and limit the surgical time and the manipulation of the surrounding tissues.^2^, ^10^, ^12^, ^23^, ^25^, ^30^ The second aim of the study was therefore to evaluate whether any intraoperative assistance technique could reduce the complication rate compared to the unassisted lymphadenectomy.

The overall incidence of postoperative complications reported in this study was 7.5%, none of which was severe or fatal. This incidence is lower than those in previous studies in which the overall complication rate ranged from 16% to 27%.^2^, ^10^, ^20^, ^24^, ^25^ This finding corroborates the assumption that peripheral LN excision is well tolerated in dogs and has a complication rate comparable with other clean surgical procedures.

The uses and advantages of intraoperative assisted techniques for lymphadenectomy have been described in several studies;^2^, ^20^, ^23^, ^24^, ^25^, ^30^ however, information on the influence of the intraoperative assistance on surgical complication rate is lacking. In a recent study, the overall incidence of complications following 245 sentinel lymphadenectomies guided by intraoperative γ‐probing and MB dye in 113 tumor‐bearing dogs was 21.2%; 91.7% of reported complications were classified as minor.^25^

As far as the authors are aware, this is the first study to compare unassisted lymphadenectomy with assisted lymphadenectomy using combined peritumoral injection of MB and γ‐probe signal and MB alone in a large cohort of dogs. It may be argued that MB is not a real assisting technique for this purpose as it is not visible through the intact skin; however, once the lymphocentrum is located by preoperative techniques, MB allows the blue lymphatic vessels to be followed to the LN, through the dissected skin over the lymphocentrum, thus speeding up the process and limiting the amount of dissection, especially in case of small LNs in difficult locations.

The results of this study unexpectedly revealed that the use of any intraoperative assistance did not reduce the complication rate compared to unassisted lymphadenectomies. The authors suggested that the low overall complication rate may have influenced the statistical analysis, leading to no significant difference between the guidance techniques. These findings align with those of Rossanese et al. (2022),^2^ despite slight differences in the intraoperative assistance techniques between the two studies. Specifically, Rossanese and colleagues compared peripheral unassisted lymphadenectomies with those assisted by MB or anchor wire, and found no significant difference.

Gariboldi et al. (2023)^12^ reported an increased detection rate of the SLNs from 90% to 95% when a γ‐probe was combined with MB, following LS; this figure was higher than that reported for MB alone or other guiding techniques.^2^, ^10^, ^23^, ^24^ Similar results were described in the study by Beer et al. (2022),^24^ which compared near‐infrared fluorescent image‐guided LN dissection with locoregional lymphadenectomies without intraoperative guiding techniques. In the current study the overall efficacy of the assisting techniques in finding all the LNs was not investigated, as only cases where lymphadenectomy was completed were included.

Overall, 15 complications were reported; they were classified as grade I according to LeBlanc et al. (2021)^29^ in 80% (12/15) of cases; all were self‐limiting and resolved after a median of 4 days. The remaining three complications were categorized as grade 2; two cases of seroma required fluid drainage and a prolongation of the NSAIDs therapy, and one dehiscence was treated by the application of skin staples without the need for a revision surgery under general anesthesia.

The third aim of the study was to assess whether the number, palpability, and the site of the SLN influenced the incidence of complications. Considering the presence or absence of surgical complications as the dependent variable, for all the examined variables *p* > .05. In the authors' opinion, this result could be related to the overall low number of complications observed and to the low number of LNs removed from each lymphocentrum (only in 7.9% of the cases more than two LNs were excised).

The rate of complicated surgeries was lower for palpable LN extirpation, even though there was no statistically significant difference. The LN measurement provided an overview of the population size and its relation to the animal's size, as a LN of the same dimensions could affect detectability differently in dogs of varying sizes. The excised LNs were nonpalpable in 9/14 (64.2%) and palpable in 5/14 (35.7%) of the complicated surgeries. This result could be expected because, in the authors' experience, the excision of palpable (but not megalic) LNs is performed easily regardless of the technique, whereas the extirpation of nonpalpable LNs can be more challenging, requiring a longer surgical time and leading to a higher morbidity.^2^ Interestingly, considering only the nonpalpable lymphadenectomies, no difference between assisted and unassisted techniques was found, although these results could be biased by the overall low number of complications.

Considering the low incidence of complications in our study and the low number of the surgeries that resulted in at least one surgical complication (14/201), we used a decision‐tree method to analyze the data. The probability of developing any complications was significantly dependent on the site of the lymphadenectomy. The excision of mandibular or retropharyngeal LNs had a 22.2% probability of developing at least one complication compared to 5.2% for the other superficial LNs. This complication rate was in accordance with that reported in previous studies.^31^, ^32^ These results are probably related to the anatomy of the neck, which is characterized by great tissue laxity and the presence of critical structures (e.g., nerves, blood vessels, salivary glands), which may lead to a wider surgical exposure and tissue manipulation to reach these lymphocenters, resulting in greater dead space, in which fluids could accumulate. The LT was also related to the development of postoperative complications in case of surgery in this anatomic site. In particular, a cutoff value of 21.5 min was found to be the most predictive factor. Seroma was the most common complication observed in this study, although it was not frequently encountered. Several hypotheses have been proposed regarding the etiology of seroma formation, including an acute inflammatory response, increased fibrinolytic activity, decreased fibrinogen levels, the presence of dead space, surgical technique, and the use of surgical devices.^33^, ^34^ However, the exact underlying mechanisms remain unclear. In a study by Chiti et al. (2023),^25^ seroma was also one of the most frequent postoperative complications following SLN removal but no specific cause was identified. It is possible that the physical activity of the dog postsurgery may contribute to seroma formation. Further research is needed to examine more comprehensively all the potential factors contributing to seroma formation following SLN removal in dogs.

In human medical literature, lymphoedema of the arms and legs, seroma and infection are the most common complications following lymphadenectomy.^35^, ^36^, ^37^, ^38^, ^39^ In a recent study on the complication rate associated with the number of LNs excised through two different skin incisions in inguino‐femoral lymphadenectomies for vulvar cancer in humans, the surgical technique that allowed for the extirpation of the largest number of LNs was the one with the higher rate of major (grade 3) complications.^40^ As reported in previous veterinary studies,^2^, ^25^, ^41^ in the present study, no correlation between the number of harvested LNs and the likelihood of experiencing at least one complication was demonstrated. This is probably due to the lower number of LNs excised from a single lymphocentrum in dogs in comparison with human beings.^42^, ^43^

Lymphoedema was reported in three cases in this study, two of which included the bilateral extirpation of inguinal LNs in a dog. Lymphoedema was always temporary and reversible, in accordance with the results reported in previous veterinary reports.^2^, ^25^ Although lymphedema resolved spontaneously, it may still have caused some degree of discomfort for the animal, as happens in human beings. For this reason, the authors considered it a complication. All the three dogs that experienced lymphoedema healed spontaneously within 7 days, which differs from findings reported in human patients.^44^, ^45^ These findings support the fact that lymphedema is probably related to the extirpation of all the lymphocenters draining a specific area. This was the reason behind the exclusion of those cases where this event occurred in our series (e.g., concurrent bilateral extirpation of mandibular and retropharyngeal LNs in dogs with oral melanoma). The main difference between lymphedema reported in this study and human lymphedema may be the greater extent of lymphadenectomies and number of LNs excised in human beings.^45^

Nerve injuries have never been reported in cases of axillary surgery, although axillary paresthesia is a very common complication in human patients.^46^, ^47^ Neuropathic pain is difficult to assess in dogs, so only the alterations of the main reflexes of the thoracic limb were assessed in case of axillary lymphadenectomy. Mild lameness (grade 1 and 2) was reported in three dogs that underwent axillary lymphadenectomy. This may be related to the wound in the axilla or stretching of nerves or muscles in this district. However, lameness resolved spontaneously within 5 days in all cases.

There was no significant difference in the incidence of complications between institutions where the lymphadenectomies were performed in this study. This is a relevant observation because it may reflect the uniformity of the surgeons' experience among the centers and, consequently, provide for better reliability and significance of these results. It may also reflect the short learning curve needed to perform this procedure safely.

One limitation of this study was its retrospective nature and the subjective assessment of the complications, which may have resulted in a nonuniform judgment of the severity of the same event. No surgeons were influenced to look for complications, potentially underestimating their incidence.

Given the low incidence of complications associated with lymphadenectomy, a larger study population is needed to better compare complication rates between assisted and unassisted LN excision and assess statistical significance. A selection bias cannot be ruled out, as the different assistance techniques were not randomly decided but depended on available technology (e.g., LS) and surgeon preference. Due to the retrospective nature of the study, it was not possible to determine whether a specific anatomic location of the LN had an impact on the choice of additional staining/localization techniques during surgery, as this information is not available in the operative reports. Lymphoscintigraphy and intraoperative γ‐MB dye were also utilized exclusively in one institution, regardless of the size and location of the LNs.

In conclusion, in this study population, the lymphadenectomy of superficial LNs in dogs was associated with a low rate of self‐limiting mild complications that resolved in few days, independently of the assistance technique applied. The complication rate among the different techniques was not statistically significant; thus, the first hypothesis of this study was rejected. Mandibular and retropharyngeal lymphadenectomies that lasted more than 21.5 min were more prone to develop at least one complication. No other factors seem to influence the outcome of the procedure.

Further prospective, randomized clinical trials are required to compare assisted and unassisted lymphadenectomies of nonpalpable LNs, and to better analyze the effect of the site of the LNs removed and the surgical time on the development of complications.

Overall, lymphadenectomy of the superficial LNs is a safe procedure, easy to perform in most cases, and worthwhile for both diagnostic and therapeutic purposes. Gamma probe plus MB and MB alone are both safe techniques and should be employed when required, although their use does not fully prevent the risk of complications.

## AUTHOR CONTRIBUTIONS

Mattioli G, DVM: Contributed to the design and draft of the study, identified 30 suitable medical records, compiled and interpreted all data, contributed to surgical management of the cases, and discussed and revised the manuscript. Cino M, DVM, PhD: Contributed to writing the work, acquisition of 15 suitable medical records, data analysis and interpretation, surgical management of the cases and discussion of the paper, and read and approved the final manuscript. Stefanello D, DVM, PhD: Contributed to the acquisition of 40 cases, data interpretation, and surgical management of the cases. Drafted and revised the paper. Drudi D, DVM, DECVS: Analyzed data for statistical significance, interpreted data, and drafted and revised the manuscript. Morello EM, DVM, PhD: Identified 19 suitable medical records and contributed to data analysis and interpretation, surgical management of the cases, and discussion of the paper. Pisani G, DVM, DECVS: Contributed to the acquisition of 20 cases, surgical management of the cases, and writing and discussion of the paper. Chiti LE, DVM, PhD: Contributed to the acquisition of 20 cases, surgical management of the cases and reading and approving the final manuscript. Pierini A, DVM, PhD: Contributed to interpreting data, and drafted and revised the manuscript. Gariboldi EM, DVM: Contributed to the acquisition of 20 cases, surgical management of the cases, and discussion of the paper. De Zani D, DVM, PhD: Contributed to performing 80 lymphoscintigraphies, writing, and discussion of the paper. Massari F, DVM, DECVS: Revised the manuscript. Giacobino D, DVM: Identified 19 suitable medical records and drafted and revised the manuscript. Martano M, DVM, PhD: Contributed to the design of the work and data interpretation, surgical management of 20 cases, and discussion of the paper, and read and approved the final manuscript.

## CONFLICT OF INTEREST

The authors declare no conflicts of interest related to this report.

## Supporting information


**Table S1.** Signalment data of the entire population.

## Data Availability

The data that support the findings of this study are available from the corresponding author upon reasonable request.
