# Supplementary material for: Peripheral sentinel lymphadenectomy in 163 dogs: Postoperative surgical complications and comparison between intraoperative dissection techniques
Source: Vet Surg. 2025 Mar 17;54(4):766–76. doi: 10.1111/vsu.14246 (PMC12063715; doi:10.1111/vsu.14246)
Supplement: Supplementary file 1 — Table S1. Signalment data of the entire population. [file VSU-54-766-s001.docx]

**Table S1:** Signalment data of the entire population.

| Patient signalment | Frequency (n of dogs) |
| --- | --- |
| Breed  -Crossbreed  -Golden retriever  -Labrador Retrievers  -Boxer  -Jack Russel Terrier  -French bulldog  -Other (<6 dogs each) | 32  19  23  14  10  9  56 |
| Sex  -Female  -Female spayed  -Male  -Male castrated | 38  45  66  14 |
| Body condition score  3  4  5  6  7  NA | 2  41  65  22  3  30 |

Abbreviations: NA, not available; n, number
